# Tobacco Use and Its Health Effects among Professional Athletes in Qatar

**DOI:** 10.1155/2016/2684090

**Published:** 2016-11-29

**Authors:** Zaina Chaabane, Zsolt Murlasits, Ziyad Mahfoud, Ruben Goebel

**Affiliations:** ^1^Sport Science Program, Qatar University, P.O. Box 2713, Doha, Qatar; ^2^Department of Global and Public Health, Weill Cornell Medicine-Qatar, Education City, Qatar Foundation, P.O. Box 24144, Doha, Qatar

## Abstract

The objective of the study was to determine the effects of tobacco use on selected markers of health and lung function in professional athletes. A total of 108 male professional athletes participated in the study from ten ball game teams in the same sport league in Qatar (age = 26.4 ± 5.1 yrs, height = 190.6 ± 11.9 cm, and weight = 91.5 ± 16.4 kg). The athletes have been playing professionally for about 6.3 years on average. In addition to demographic and tobacco use status, the following clinical variables were measured: resting blood pressure, heart rate, FVC, FEV_1_ sec, and PEF. The prevalence of tobacco use among the athletes was 27.7%. The FVC, FVC%, and FEV_1_% were significantly lower among the smokers compared to the nonsmokers (*p* = 0.003, 0.044, and 0.001, resp.). There were no significant differences between cigarettes smokers and nonsmokers in BP, HR, FEV_1_, FEV_1_/FVC, PEF, and PEF%. Similarly, those who smoked shisha had lower FEV_1_% values as compared to those who did not smoke shisha (*p* = 0.001). The decrease of FEV_1_ and FVC among smokers compared to nonsmokers is similar to what has been reported in the literature about other populations.

## 1. Introduction

Tobacco use, especially cigarette smoking, has been associated with many diseases, such as bronchitis, stroke, cancer, and heart diseases [1]. Athletes are no exception and those who use tobacco are prone to the same dangerous health effects. Professional athletes are commonly characterized as being fit, strong, and healthy. To be able to perform at the competitive level the respiratory (e.g., lung function) and the cardiovascular systems should be in perfect health and function optimally. However, these systems are affected by smoking, impeding their normal function and leading to reduced athletic performance [2].

Physical exercise has been reported to reduce the incidence of some smoking hazards such as lung cancer [3]. Women who were smokers or former smokers but were involved in physical activities had a reduced chance of suffering from lung cancer compared to those who were inactive [4]. On the other hand, tobacco use affects the lungs, reducing their capacity and the ability to take in enough oxygen; therefore it can negatively impact an athlete's performance [5]. Athletes are known to experience effects, such as increased resting heart rate, narrowed blood vessels, and less oxygenated blood among others [3]. One of the repercussions of smoking is that it constricts the blood vessels, due to the imbalance of vasodilatory and vasoconstrictive actions originating from the endothelium [6]. The reduced vessel size makes it difficult to pump enough blood to the working muscles, which in turn can affect athletic performance. Increased sympathetic activation and heart rate, along with coronary and peripheral vessel constriction, lead to increased myocardial workload, placing greater stress on the heart [6, 7]. Moreover, the inhalation of carbon monoxide found in cigarettes reduces oxygen delivery, because CO combines with hemoglobin in the blood to form carboxyhemoglobin (COHb), displacing oxygen and also causing a leftward shift in the O_2_-Hb disassociation curve. In fact, the level of COHb found in smokers was higher compared to nonsmokers, which had a clear impact on the ability of the muscles to resist fatigue during voluntary muscle contractions [8]. Moreover, impaired exercise tolerance and exercise capacity in young smokers have been explained by reduced tissue oxygenation, arising from arterial O_2_ desaturation and insufficient O_2_ delivery [6].

Exposure to passive smoking has also been associated with similar health risks [9]. In fact, passive smoking was linked to a significant reduction in pulmonary function (forced expiratory flow 25–75%) and an increased incidence of cough in young athletes [10, 11]. This may point to the involvement of small airways in asymptomatic athletes, because the small airways may be more susceptible to the harmful effects of cigarettes. It has also been reported that several spirometry measures, including forced expiratory volume in the first second (FEV_1_), were significantly reduced in young athletes exposed to passive smoking compared to their unexposed counterparts.

According to a study by Kobayashi et al. [12], which analyzed VO_2max_ and heart rate in 9 young smokers and 9 nonsmokers during a maximal treadmill test, VO_2max_ was lower in smokers compared to the nonsmokers. Smoking status was also significantly related to cardiorespiratory fitness as measured by 1.5-mile endurance performance in young, healthy navy personnel [13]. Most importantly, this association was independent of other factors, such as activity level.

A study conducted by Al-Obaidi et al. [14] examined lumbar extensor strength in smoker and nonsmoker athletes and found that smokers exhibited decreased strength when compared to the nonsmoker athletes. In addition, cigarette smoker army recruits exhibited a higher rate of injuries during 8 weeks of basic training compared to nonsmokers [15].

Qatar is a fast growing country where there is a great interest in sports and in general physical activity. Qatar has hosted numerous international sporting events and will be the host of the 2022 FIFA Football World Cup. Qatar is probably the only country that has a one-day national holiday dedicated to sports. However, no studies so far had attempted to measure tobacco use and its effects on lung function in Qatari athletes.

In light of the potential effect of tobacco use on athletes, this study aimed to measure the prevalence of tobacco use among professional athletes in Qatar and to determine its effects on selected health indicators and physiological parameters, such as lung function.

## 2. Methodology

### 2.1. Subjects

113 male professional athletes from ten ball game clubs in the same sport league in Qatar were recruited between 1st of May 2015 and 31 of May 2015. Four athletes from different clubs refused to participate for personal reasons, and one player withdrew during the test for being uncomfortable with spirometry testing. A total of 108 athletes were included in the study and the final data analyses (age = 26.4 ± 5.1 yrs, height = 190.6 ± 11.9 cm, and weight = 91.5 ± 16.4 kg). Subjects characteristics are depicted in Table 1. The athletes have been playing professionally for 6.3 years on average and 50% of the participants were Arabs. The study was conducted according to the Declaration of Helsinki for the protection of human participants and approved by the Local Ethics Committee at Qatar University before commencement. A consent form was also signed by each athlete before participation. The questionnaire and all testing were administered by the same trained technician to avoid intertester variability and minimize experimental errors.

### 2.2. Questionnaire

The athletes were asked to come one hour before their practice session to discuss the study and sign the informed consent prior to testing. Athletes went through three phases of the project which are as follows: in phase one, the athletes read and signed the informed consent and made sure that everything was clear to them and could ask any question before they moved to next phase. In phase two, they filled out a questionnaire related to their smoking habits and opinion and knowledge about tobacco; the questionnaire was in English language and contained 22 close ended questions regarding demographics, tobacco use, knowledge about the harmful effects of tobacco on sport performance, and attitudes towards sportspeople tobacco use and banning of tobacco use in public places. A technician was available to clarify the questions or to translate some points that the athletes could not understand. In phase three, anthropometric measurements were conducted on the athletes and then following five minutes of rest, resting BP and HR were recorded followed by the spirometer test.

### 2.3. Lung Function Testing

A spirometer was used (Vicatest P2a, Mijnhardt, Holland) to measure lung function, such as FVC, FEV_1_, and PEF. The test was conducted in a seated position, with each athlete using a nose clip and placing the mouth piece in the mouth making sure that the lips were placed in a way that no air could enter and then following a maximum inhalation, exhaled as hard as they could until they felt that no air left in their lungs. Each athlete had the chance to practice the protocol prior to testing. The actual test consisted of three attempts, of which the best result was recorded and used for statistical analyses.

### 2.4. Data Analyses

Demographic, health, and tobacco related variables were summarized using means and standard deviations for numeric variables such as age and height and frequency distributions for categorical variables such as tobacco use. The prevalence of cigarette smoking as well as shisha smoking was computed along with their 95% confidence intervals. Independent* t*-test was used to detect differences in BP, HR, and lung function variables between tobacco users and nonusers and also for cigarette smokers (daily or occasionally) and nonsmokers. Similar analysis was done for comparing those who smoked shisha to those who did not. A* p* value of 0.05 was considered statistically significant. All analyses were conducted using IBM-SPSS version 23.

## 3. Results

The prevalence of smoking cigarettes (daily or occasionally) was 11.2% (95% confidence interval 5.2%–17.2%) and that of smoking shisha (daily or occasionally) was 22.4% (95% confidence interval 14.5%–30.3%). In addition, about 5% of the participants reported smoking cigars or Medwakh. 38.9% of the participants reported having a father who uses tobacco. The vast majority of the athletes knew that cigarettes and shisha are bad for health (90.7% and 85.2%, resp.), but only 54.6% of them reported that tobacco use has a negative effect on the athlete's performance. 81.5% of the athletes agreed with a total ban on tobacco use in all indoor public places in Qatar, while 58.3% of the athletes were against professional athlete's using tobacco.

Participants were grouped into two categories: smokers (cigarettes, shisha, cigars, or Medwakh) and nonsmokers. Smokers had significantly lower mean values of FVC, FVC%, and FEV_1_% as compared to nonsmokers (*p* values = 0.003, 0.044, and 0.001, resp.) (Table 2). No other measured variable differed significantly between the two groups. Moreover, there was no significant difference between cigarettes smokers and nonsmokers in terms of BP, resting HR, FEV_1_, PEF, and PEF%. Similarly, those who smoked shisha had lower FEV_1_% values compared to those who did not smoke shisha (*p* = 0.001) (Table 3). No other health indicator among those tested was significantly different between those who smoked shisha and those who did not.

## 4. Discussion

Professional athletes are commonly characterized as being fit, strong, and healthy; thus smoking is usually not thought of as significant issue in professional sports. However, considering the widespread tobacco use in the Middle East, professional athletes are also exposed to the unhealthy habit of smoking. The prevalence of tobacco use among athletes in our study (27.7%) seems to be comparable and even higher than that of the general Qatari male population (21.1%) [16]. This is an alarming finding in Qatar, considering that studies generally report lower smoking rates in athletes in the world, including the Middle East [17, 18].

Although the knowledge about the harmful effects of cigarettes and shisha smoking seems to be sufficient, still 11.2% of the athletes smoked cigarettes and 22.4% smoked shisha. Moreover, 16.7% of the athletes either thought that shisha is safer than cigarettes or reported insufficient knowledge about its effects. Given the shisha use and its health risks in the region in the past 20 years, specific health campaigns about tobacco use in general and shisha smoking in particular should be tailored to athletes, especially that these athletes are considered role models for a lot of teenagers and children. Also 21.3% of the athletes either thought that smoking has positive or no impact on the performance of the athlete or did not know what kind of effect it poses. Such lack of knowledge should be addressed in health campaigns for athletes. In fact, several studies have reported that smoking is detrimental to exercise performance as young male smokers exhibited reduced exercise tolerance [19], while the HR recovery of female smokers was impaired [20]. To support this notion Albrecht et al. [21] reported that peak oxygen consumption and exercise duration were increased to a greater extent after an exercise program in females who quit smoking compared to those who continued the habit.

In the current study we did not find a significant increase of resting HR and BP when we compared smokers and nonsmokers, which contradicts the available reports [19, 20]. This discrepancy may be explained by the different populations used. While data is currently available on healthy young adults, our study analyzed professional athletes; thus it is feasible that HR adaptations to long-term training in professional athletes counteract the HR increase seen in average smokers.

One limitation of the current study is that we could not conduct athletic performance measures, because the players were at the end of their competitive season, so smoking status and the lung function could not be associated with performance measures, such as VO_2max_ or athletic achievement. Also, bronchial hyperresponsiveness was not examined, which may have provided additional measures of smoking-related effects in athletes. Therefore, future studies should analyze performance measures and success in smoker and nonsmoker athletes to link these measures and convince athletes of the negative effects of smoking on athletic performance. In addition, the small sample size might render some difference statistically nonsignificant. However, to the best of the author's knowledge, this is the first study to analyze the effects of smoking on lung function in Middle Eastern professional athletes.

## 5. Conclusions

However, it is clear from the study results that smoking affects markers of lung function. In fact, changes in pulmonary function could be detected with less sensitive parameters of lung function, namely, FVC and FEV_1_, which mainly reflect the function of large airways. The lower FEV_1_ and FVC among smokers compared to nonsmokers are similar to what is presented in the literature; hence athletes are not immune to these health risks as these data indicate that regular strenuous physical activity does not protect the lungs from the detrimental effects of smoking. It has been reported that even passive smoking leads to lower pulmonary function in young athletes [10, 11]. Moreover, college students exhibited reduced FVC and FEV_1_ immediately after smoking two cigarettes [22]. Studies support the harmful effects of tobacco use in sedentary populations and in professional athletes not only on general health, such as pulmonary and cardiovascular functions, but also on athletic performance. Athletes are important public figures and considered role models; hence, athletes should be informed about the health risks of tobacco use and its effects on their performance. Moreover, cessation programs should be promoted and put in place to help athletes quit.

## Figures and Tables

**Table 1:** Sample characteristics.

Variable	Mean ± SD	Smokers	Nonsmokers
	*N* (%)	(*N* = 30)	(*N* = 78)
Age (years)	26.4 ± 5.1	26.0 ± 5.2	26.5 ± 5.0
Height (cm)	190.6 ± 11.9	191.0 ± 10.6	190.4 ± 12.4
Weight (kg)	91.5 ± 16.4	91.5 ± 16.3	91.4 ± 16.6
Nationality			
Arab *n* (%)	54 (50.0%)	21 (70.0%)	33 (42.3%)
Non-Arab *n* (%)	54 (50.0%)	9 (30.0%)	45 (57.7%)
Years of professional playing			
0–2	30 (27.8%)	9 (30.0%)	21 (26.9%)
3–5	29 (26.9%)	8 (26.7%)	21 (26.9%)
6–8	17 (15.7%)	2 (6.7%)	15 (19.2%)
≥9	32 (29.6%)	11 (36.7%)	21 (26.9%)
Mean ± sd years	6.3 ± 4.7	6.7 ± 5.2	6.1 ± 4.5

**Table 2:** Association between tobacco use and health indicators.

Variable	Any tobacco use		*p* value
	Yes^†^	No	
	(*N* = 30)	(*N* = 78)	
	Mean ± sd	Mean ± sd	
SBP (mmHg)	135.9 ± 7.4	134.6 ± 9.4	0.515
DBP (mmHg)	76.4 ± 8.8	75.8 ± 9.6	0.768
HR (bpm)	66.7 ± 11.7	67.4 ± 10.5	0.775
FVC (L)	4.20 ± 0.81	4.57 ± 0.78	0.030^*∗*^
FVC%	73.9 ± 14.2	79.6 ± 12.6	0.044^*∗*^
FEV_1_ (L)	3.64 ± 0.90	3.97 ± 0.75	0.056
FEV_1_%	72.4 ± 16.3	84.6 ± 12.8	0.001^*∗*^
FEV_1_/FVC	0.88 ± 0.18	0.87 ± 0.12	0.916
PEF (L)	7.17 ± 2.92	7.56 ± 2.57	0.504
PEF%	67.6 ± 27.3	72.1 ± 26.3	0.443

^*∗*^Significant difference at 5% level.

^†^Yes above also includes cigar, Medwakh.

SBP: systolic blood pressure, DBP: diastolic blood pressure, HR: heart rate, FVC: forced vital capacity, FEV_1_: forced expiratory volume in 1 sec, PEF: peak expiratory flow, FVC%: % of predicted forced vital capacity, FEV_1_%: % of predicted forced expiratory volume in 1 sec, and PEF%: % of predicted peak expiratory flow.

**Table 3:** Association between tobacco use and health indicators.

Variable	Cigarette smokers		*p* value	Shisha smokers		*p* value
	Yes	No		Yes	No	
	(*N* = 12)	(*N* = 95)		(*N* = 24)	(*N* = 83)	
SBP (mmHg)	138.0 (7.2)	134.7 (9.0)	0.227	135.8 (7.6)	134.9 (9.2)	0.636
DBP (mmHg)	74.8 (12.5)	76.2 (9.0)	0.624	76.5 (9.0)	75.9 (9.5)	0.791
HR (bpm)	68.6 (11.6)	67.1 (10.7)	0.657	67.0 (12.5)	67.3 (10.3)	0.907
FVC (L)	3.82 (0.72)	4.55 (0.78)	0.003^*∗*^	4.37 (0.78)	4.50 (0.82)	0.492
FVC%	69.0 (14.8)	79.2 (12.8)	0.012^*∗*^	75.6 (12.9)	78.8 (13.5)	0.304
FEV_1_ (L)	3.50 (0.84)	3.92 (0.80)	0.088	3.68 (0.97)	3.93 (0.75)	0.172
FEV_1_%	69.0 (16.2)	82.6 (14.0)	0.002^*∗*^	72.6 (16.8)	83.5 (13.3)	0.001^*∗*^
FEV_1_/FVC	0.93 (0.18)	0.87 (0.13)	0.156	0.85 (0.19)	0.88 (0.12)	0.420
PEF (L)	7.32 (3.38)	7.45 (2.59)	0.873	6.81 (3.04)	7.61 (2.54)	0.194
PEF%	69.3 (30.9)	70.8 (26.2)	0.848	64.1 (28.4)	72.5 (26.0)	0.172

^*∗*^Significant difference at 5% level

SBP: systolic blood pressure, DBP: diastolic blood pressure, HR: heart rate, FVC: forced vital capacity, FEV_1_: forced expiratory volume in 1 sec, PEF: peak expiratory flow, FVC%: % of predicted forced vital capacity, FEV_1_%: % of predicted forced expiratory volume in 1 sec, and PEF%: % of predicted peak expiratory flow.

## Abbreviations

BP: Blood pressure
HR: Heart rate
FVC: Forced vital capacity
FEV_1_: Forced expiratory volume in 1 sec
PEF: Peak expiratory flow
FVC%: % of predicted forced vital capacity
FEV_1_%: % of predicted forced expiratory volume in 1 sec
PEF%: % of predicted Peak expiratory flow
VO_2max_: maximal oxygen uptake.
